# A Model of Health for Family Caregivers of Elders

**DOI:** 10.3390/healthcare5010001

**Published:** 2016-12-22

**Authors:** Florence M. Weierbach, Yan Cao

**Affiliations:** College of Nursing, Graduate Programs, East Tennessee State University, Johnson City, TN 37614, USA; caoy01@etsu.edu

**Keywords:** family caregivers, health, elders, model, longitudinal study

## Abstract

Family members who provide care to their loved ones experience changes in their own health. The caregiver health model (CGHM) is a new model that identifies health holistically and identifies four determinant(s) that contribute to the health status of the family caregiver. The purpose is to introduce the CGHM: Hypothesis 1: the determinants of health in the CGHM contribute to the health of the Caregiver, Hypothesis 2: the determinants of health contribute to changes in the caregivers’ health at 8 and 16 weeks, and Hypothesis 3: a change in health occurs from baseline to 8 and 16 weeks. Methods: A descriptive, longitudinal design used three data collection points and five survey instruments. Community recruitment (N = 90) occurred through word of mouth and newspapers. Inclusion criteria consisted of being a family caregiver, living in a rural residence, and providing care to elders with necessary activities of daily living (ADLs) and/or instrumental ADLs (IADLs). Following a participant generated phone call to provide consent, caregivers received an initial study packet, additional packets were sent upon return of the previous packet. Analysis for the three hypotheses included multiple backwards stepwise linear regression, generalized estimating equations (GEE), and analysis of variance (ANOVA) α = 0.05. Results: A significant decrease in mental (*p* < 0.01) but not physical health at 8 weeks (*p* = 0.38) and 16 weeks (*p* = 0.29) occurred over time. Two determinants displayed significant (*p* < 0.05 or less) changes in mental and/or physical health at one or more time points. Study limitations include caregiver entry at varying times and self-report of elder nursing needs and medical conditions. Conclusions: Findings support two of the four determinants contributing to caregiver health.

## 1. Introduction

In recent years, roughly 43 million family members in the U.S. [1], 14% of whom live in rural areas have provided care to community dwelling elders [2]. In the U.S., the average family caregiver provides 20 h of care a week in informal care to community dwelling older adults [1], with caregivers who provide care for 21 h or more considered high-intensive caregivers [3]. The care the family member provides is multifaceted, and has significant consequences on the family member. Caregivers perform or assist with activities of daily living (ADLs) [4] and instrumental ADLs (IADLs) [5]. Caregivers also provide nursing care, which includes medication administration, tube feedings, wound care, monitoring blood pressure or blood sugar, and operating medical equipment such as oxygen [3]. Financial assistance is provided by caregivers, with almost half (42%) of them spending $5000 a year or more of their own money on expenses related to the care they provide to their loved ones [1].

Care that is provided to community dwelling elders by family members impacts the family caregivers’ physical and mental health. Family caregivers report fair to poor health and multiple chronic conditions. Approximately 75% of caregivers report having five or more chronic conditions [6]. The five most common conditions reported include hypertension, high cholesterol chronic back pain, heart disease, and arthritis [6]. When family members assume the caregiver role, they are particularly vulnerable to physical exhaustion and emotional constraints [7]. Spousal caregivers perceive worse physical health and depressive symptoms than other family caregivers [8]. In addition to the physical health impact on the family caregiver, there are mental health implications with caregivers having higher rates of depression and feelings of isolation and invisibility, and additional impacts include a loss of self, time, and money and almost twice as many physician visits when compared to non-caregivers, with more than 40% of caregivers dying before the care recipient [9]. While family caregivers have poor health, it is not clear how they view their health and what they believe contributes to their health. It is important to consider what family caregivers identify as influencing their health both positively and negatively.

**Figure 1:**
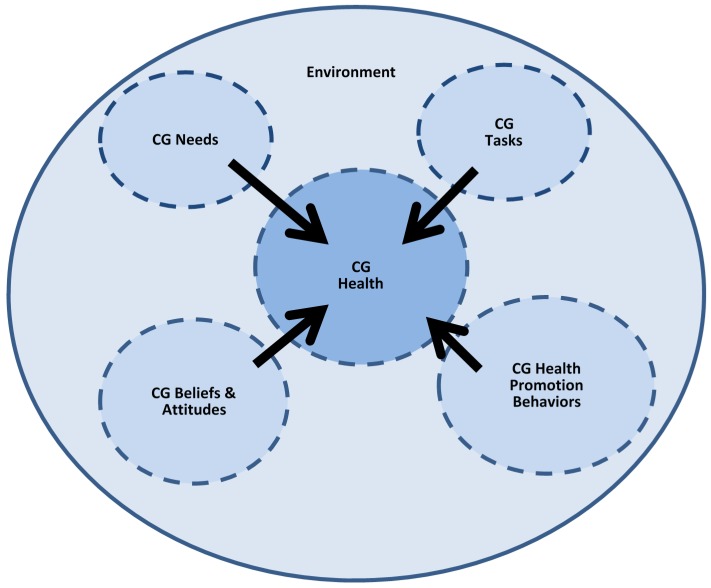
Caregiver health model (CGHM).

The purpose of this discussion is to introduce and test the caregiver health model (CGHM) (Figure 1). The CGHM is a comprehensive model for family caregiver health that addresses health holistically. The CGHM places the family caregiver within the context of the geographic location of where the care is provided. It identifies five constructs: the dependent construct is health, with the four independent constructs referred to as determinants. Health is operationalized holistically based on the World Health Organization (WHO) definition [10]. The four remaining variables are identified as determinants, which include, caregiver health promotion activities, caregiver attitudes and beliefs, caregiver tasks, and caregiver needs.

The CGHM was developed after a review of caregiver health literature focusing on the care provided by caregivers in the U.S., which has a health care system that focuses predominantly on secondary prevention and individual health care needs, not on primary and tertiary care or the needs of the family caregiver. The broad search included CINAHL and PubMed databases with the following key words: elder, geriatric, health, caregiver, stress, and burden. An ancestral approach was employed to capture caregiver literature that was not identified with the databases.

What family members do to maintain their health varies. It is unfortunate and not unusual that in the process of caring for their family members, caregivers often develop health problems of their own which they may ignore [11]. Caregivers experience changes in their health status while providing care for their loved ones. The earliest change in caregiver health identified in U.S. literature is with caregivers of patients with cardiac disease [12]. The first study time after enrollment was 16 weeks, which showed a change in the health of the caregiver [12]. In addition to the length of time as a caregiver, higher levels of caregiving have also been found to impact caregivers’ mental health, physical health, and health-risk behaviors [13]. Caregivers limit their view of health promotion to nutrition and exercise; they do not consider tobacco, drug, or alcohol as behaviors that need attention [14].

How family members view caregiving impacts their health. Family caregiver beliefs and attitudes have been shown to negatively affect health outcomes [15]. In a qualitative study of female caregivers, who provided care due to obligation and without emotional involvement, the caregivers experienced feelings of ambivalence and poorer psychological health [16]. In contrast, when the relationship of caregiver and care recipient was positive, caregiver health outcomes improved [15]. Obligations of family members to provide care are identified as cultural traditions and expectations. For some individuals and cultural groups, the expectation and provision of care may be viewed as a source of happiness [17].

The tasks caregivers do and the unmet caregiver needs have been found to negatively impact health. Caregivers assist elders with a multitude of tasks. It is not unusual for family caregivers who provide care by completing tasks to experience burden. The manifestation of burden by the caregiver is frequently viewed as having a negative impact on health, which is manifested as caregiver stress. The caregivers’ perception of what they need help with is important to consider and is impacted by caregiver stress, patient problem behaviors, and caregiver workload [18]. Caregivers’ needs are based on the care recipient’s disease and disease management skills, home modifications, and the provision of supplies in the home. Consideration of the caregiver’s own personal, physical, spiritual, and psychological needs, however, often take a back seat to the care recipient’s needs by the family caregiver and professional nurses [19,20,21,22,23,24]. Where the caregiver lives may impact the needs of the caregiver and their ability to have help with the tasks they provide for the elder.

Comparing health between two geographic locations, such as rural and urban, is common practice. Defining rural as non-urban or using specific racial groups in rural settings may limit the application of the findings to different classifications of rural that are present with rural classification schema [25]. The use of broad definitions of rural, focusing on specific ethnic and racial groups, limits knowledge related to the rural family caregiving experience [26]. Rural caregiving studies that address cultural groups located in isolated rural areas in the U.S. are limited. Rural residents face challenges in accessing health and social services, which may further impact the ability of the caregiver to receive assistance in providing care to their family member. Within Appalachia, 42% of the population lives in areas designated as rural. Health care resources are limited, not well coordinated, and, in some rural areas, health care may be non-existent [27]. In addition to limited health care, access to the internet and cell service for communication is also limited. In South Central Appalachia (SCA), the residents consider themselves culturally as Appalachian with the majority of the counties classified as rural [27]. Having an understanding of how the geographic locations contribute to the similarities or differences in the findings may not be clearly understood.

## 2. Research Method 

The research design for testing the CGHM was a descriptive, longitudinal design with data collection at three points: baseline, 8 weeks, and 16 weeks. Hypothesis 1: The determinants of health in the CGHM contribute to the health of the caregiver. Hypothesis 2: The determinants of health contribute to changes in the caregiver’s health at 8 and 16 weeks. Hypothesis 3: A change in health occurs from baseline to 8 and 16 weeks.

Instruments: Health and the four determinants of health were measured with valid and reliable instruments (Table 1) which were self-administered and relied on self-report. Health, the dependent variable, was measured with the National Institutes of Health (NIH) Promis Global Health instrument which measures four dimension of health: Physical Health (PH), Mental Health (MH), Social Health (SH), and Global Health (GH) [28]. Determinants of health, the independent variables, were measured with four instruments: Walker’s Health Promoting Lifestyle Profile II (HPLPII) [29], Kosloski’s Caregivers’ Beliefs and Attitudes Scale [30], Oberst’s Caregiving Burden Scale [31], and Hileman’s Home Caregiving Needs Survey [32].

All five of the instruments use a Likert-type scale. Hileman’s home survey has three choices for each question; the weighted score for each question of Hileman’s home survey is determined by the responses from the three choices for each question [32]. See Table 1 for instrument information.

Sample: Family caregivers were recruited from the SCA region [27]. Recruitment was accomplished with the assistance of senior centers, area agencies on aging, health care organizations, and local print media, which included press releases, feature articles, and paid advertising. Participants contacted the primary investigator by phone or email. When the initial contact was by email, the primary investigator arranged a time to talk with the potential participant by phone. During the phone meeting, the study was explained to the participant. If the participant met the inclusion criteria and agreed to be in the study, verbal consent was obtained.

Inclusion criteria included self-identification as a family caregiver for a community rural dwelling elder 55 years or older who required assistance with two or more activities of daily living (ADLs) [4] or instrumental activities of daily living (IADLs) [5]. All participants lived in SCA and considered the area where they lived to be rural. Individuals were eligible to be in the study regardless of the length of time they had been providing care. The majority of caregivers reported they had a chronic health condition and provided greater than 90 h of care a week. Table 2 provides participant characteristics by gender for Time 1.

Ethical Approval: Human subject approval was obtained from the East Tennessee State University Medical primary investigator’s university Institutional Review Board (IRB) (Project identification code: 1010.8sd), and from Mountain City Health Alliance and Wellmont Health System, the two local health care organizations located in the SCA. IRB approval was granted to complete the study with the procedure protocol designed to answer the three hypotheses. The protocol included an informed consent statement which stated the risks and benefits of participating in the study. Participant recruitment occurred after IRB approval was obtained.

Study Procedures: The first packet of instruments (baseline) with a copy of the phone consent form was mailed to the participant. When the baseline study packet was returned, the second study packet was mailed 6 weeks later and the third study packet was mailed 6 weeks after the second study packet was returned. All study packets included a self-addressed envelope with postage paid, the five instruments, and either the data base (baseline) or data base update sheet (Time 2–3), which allowed the participant to update information regarding themselves or the care recipient. Participants were paid a total of $15 for completing instruments at all three time periods; the majority of participants (N = 50) did not want or receive reimbursement.

Attrition occurred over the three time periods. Recruitment yielded 90 family caregivers who consented to participating in the study, with 62 (68.8%) completing the entire study. The participants who completed Time 1 were committed to the project, which is demonstrated by 17.4% attrition from Time 1 to Time 3, which is lower than a 24% return rate (76% attrition) with a longitudinal postal study with three time points [33].

No packets were returned to the PI due to an incorrect address. One participant asked to be removed from the study after consenting and receiving the baseline packet; the participant did not complete the baseline packet and was removed from the study. The majority of caregivers reported they had a chronic health condition and provided greater than 90 h of care a week. Table 2 provides participant characteristics by gender at baseline.

### 2.1. Data Analysis Plan

Data entry occurred as packets were received from the participants. For accuracy, at the completion of the study data entry was verified by a second person who reviewed each participant’s responses by time period. After the data were verified, the data were cleaned and assessed for missing data with less than 10% missing data for each instrument item for each time period. SPSS version 22 (IBM Crop., Armonk, NY, USA) was used for all statistical analysis. Prior to analysis, the data set was assessed for descriptive statistics and frequency distributions for each demographic item, instrument item, subscale, and time period. Collinearity was tested on all survey instruments, based on the variance inflation factor (VIF), and collinearity was detected only between needs subscales. One needs variable was calculated based on 6 categories of needs variables to eliminate collinearity. Alpha level was set at 0.05 for analysis using multiple stepwise backwards regression and generalized estimating equations (GEEs). Reverse coding was completed on individual questions with the PROMIS instrument that were part of the PH subscale. The data set contained scores on instrument subscales that were computed and analyzed for internal consistency and reliability with Cronbach alpha at baseline (Table 1).

### 2.2. Analysis and Findings

Hypotheses 1, 2, and 3 utilized three analysis methods. Hypothesis 1 used multiple stepwise backwards linear regression analysis to determine the significant contributors to PH and MH for each time period (Table 3). The variables that are significant contributors in the final model for each time are listed in Table 3. Health promotion and spiritual growth was the only measure that was significant for both PH and MH at all three times. Spiritual growth had a positive relationship with PH (baseline: 0.596; 8 weeks: 0.406; 16 weeks: 0.479) and MH (baseline: 0.305; 8 weeks: 0.386; 16 weeks: 0.351) at each of the three times. Needs was significant for MH with a negative relationship (baseline: −0.03; 8 weeks: −0.05; 16 weeks: −0.03) at all three times. Health promotion and physical activity was significant for PH at baseline (0.343) and 16 weeks (0.375). Physical health increased as physical activity increased. Variables about caregivers’ beliefs and attitude, and variables about caregiving demand and difficulty were not significant for PH and MH at any of the three time points.

Hypothesis 2 used generalized estimating equations (GEEs) to determine the significant contributors to PH and MH longitudinally that account for within-subject correlation in mental health or physical health over time. Based on an examination of the data, we assumed that the structure of the within-subject correlation was autoregressive, i.e., AR (1). GEEs model used physical health or mental health responses as dependent variables. The independent variables of interest were sevenfold (Health Responsibility, Physical Activity, Nutrition, Spiritual Growth, Interpersonal Relations, Stress Management, and Needs) and were significant in the linear regression model at each time. See Table 4 for the predictors associated with PH and MH longitudinally. Longitudinal models were run for the 7 measures with repeated measures at three time points: baseline, 8 weeks, and 16 weeks. From baseline to 8 weeks, and from 8 weeks to 16 weeks, both PH score and MH score decreased, but MH score decreased significantly. The determinant measures which were significant or marginally significant positive contributors for PH include Health Responsibility (*p* = 0.04), Physical Activity (*p* < 0.01), Nutrition (*p* = 0.03), and Spiritual Growth (*p* = 0.02). Needs (*p* = 0.05) was a negative contributor to PH. A determinant measure that was significant or a marginally significant positive contributor for MH was Spiritual Growth (*p* = 0.04), while Needs (*p* < 0.01) was a negative contributor.

Hypothesis 3 was tested using an Analysis of Variance (ANOVA) and a Bonferoni post hoc test to determine which time period had a change in health for MH. The only change in health was with MH between baseline and 16 weeks. A change in MH was present between baseline and 16 weeks. No change in MH was present from baseline to 8 weeks. No change in PH was present for any time period. See Table 5.

## 3. Discussion

Hypothesis 1 findings indicate that the determinants of health vary based on the two health dimensions, PH and MH. Measures from two of the four proposed determinants of caregiver health contribute to PH or MH. The health promotion and needs determinant were found to contribute to PH and MH at each time period and for the change in health over time. Four of the health promotion subscales and the needs variable contributed to either PH or MH for one or more of the three time periods. The lack of the task, attitude, and belief determinants contributing to PH and MH is unexpected. Addressing tasks and needs as separate variables in the model may account for the change in caregiver PH and MH, with needs being stronger than tasks.

The tasks the caregiver performs and their needs vary based on their abilities. Identifying needs as determinants that contributes to health in the CGHM provides the caregiver a mechanism to have their needs acknowledged. The lack of the task, attitude, and belief variables contributing to PH and MH may be due to the inclusion criteria for the caregivers being on ADLs and IADLs rather than a medical diagnosis of Alzheimer’s disease. Knowledge of which specific subscale related to health promotion (health responsibility, physical activity, nutrition, spiritual growth, interpersonal relations, and stress management) contributes the most to PH and MH is important considering the available health care resources in rural communities [34].

Spirituality was a subscale in three of the four survey instruments in the CGHM. Two of the subscales, one in Hileman’s Home Caregiving Need Survey (spiritual needs) and one in Kosloski’s Measure of Caregivers Beliefs and Attitudes (Attitudes regarding help: Spirituality), did not show any contribution. In Walker’s HPLPII, however, spiritual growth contributes to health. With caregivers identifying spiritual activities less than 10% of the time as health promotion activities [13], the contribution of the HPLPII spiritual growth subscale is an important finding. Historically, the residents of the geographic location of the study have viewed religion as important [35,36]. In 2015, the city in the middle of the area where recruitment occurred was listed as the third most Bible-minded city in America [37]. The importance of religion in the region may explain why spiritual growth is a positive contributor to PH and MH. While spirituality and religion are two separate concepts [38], it is not known how the individual caregiver participants view spirituality and religion. This needs further exploration.

Hypothesis 2 findings indicate the variables that contribute to caregiver PH and MH changes over time, with the two dimensions of health, PH and MH, representing a holistic view of health based on the WHO definition of health [10]. Having an understanding of which determinants, and which specific determinant variables of health, contribute to health provides a new understanding of what the caregiver views as contributing to their health.

Hypothesis 3 findings show changes in MH but not PH over time. An earlier study that did not include an 8-week time point identified changes in physical health at 16 weeks [12]. MH worsens between baseline and 16 weeks with no change between 8 weeks and 16 weeks. Having an understanding of whether change occurs in caregiver PH and/or MH at specific time points (8 and 16 weeks) allows health care professionals to develop and offer programs to caregivers that will assist them in maintaining or even improving their own health while providing care to their loved one. Focusing on measures that contribute negatively to PH and MH encourages the development of interventions to enhance or alleviate the negative contribution the measure has on PH and/or MH.

## 4. Limitations & Nursing Implications

The number of participants who completed all three time periods limits the findings. The lower than anticipated number of participants decreases the effect size, power, and impact of the findings. The lack of specificity of the length of time the family member provided care prior to study entry is a limitation of the study. However, given the recruitment challenges of the rural environment and isolated caregivers, inclusion criteria addressing caregiving time would have extended the overall length of the study. Participants were recruited through referrals from health care and geriatric care providers, word of mouth, and the media. While health care and geriatric care providers referred some participants, the majority of the participants contacted the PI after reading about the study in the newspaper or because a friend told them about the study. A concern about the study was the lower number of participants who were referred by health care and geriatric professionals.

Research addressing caregiver health needs from the care recipients ADLs and IADLs rather than their medical diagnosis is gathering interest. A recent survey included questions addressing ADLs, IADLs, and medical/nursing tasks the caregiver provides for the care recipient [3]. The inclusion criteria for the caregiver in the CGHM study did not consist of the medical diagnosis of the care recipient; rather, it was the amount and type of ADL or IADL that the caregiver provided for the care recipient [4,5]. The instruments measuring the concepts in the caregiver health model were based on the care recipients ADL/IADL needs—not on their medical diagnosis. Three of the five instruments, Kosloski’s measure of caregivers’ beliefs and attitudes, Hileman’s home caregiving need survey, and Oberst’s caregiving burden scale, were developed and used on caregivers of patients with either Alzheimer’s or cancer [29,30,31]. Extending the use of the three instruments to this population of caregivers who met the criteria for the study based on the type of assistance they provided their loved ones demonstrates the utility of the application of the instrument beyond a medical diagnosis.

The CGHM provides a framework which may assist health care professionals in making decisions to promote caregiver PH and MH. The findings from the study contribute to the literature by providing information about what the caregivers recognize as major contributors to PH and MH. The self-report and perceptions of the caregivers grant the opportunity for examination and expression of thoughts about what they do for their loved ones and how they care for themselves. Future studies need to address and incorporate different environments, such as urban, suburban, and other regions of the country. Further exploration of the six measures of Walker’s HPLPII [29] will assist in determining which specific measures contribute the most to PH and MH. Adding social health in future studies will expand the utility of the CGHM to include other human service disciplines, such as social work.

The SCA region has rural communities which are isolated due to geography. Within the region and the isolated communities are multiple Christian sects, such as “Mountain Churches and Free Will Baptist”, which are not present in other rural areas [35]. In addition to the churches that are specific to the region, multiple religious groups representing Baptist, Methodist, and Presbyterian traditions are present [36]. In 2015, the study regions were home to the second, third, and fourth most Bible-minded cities in the country [37]. Spirituality as a contributor to PH and MH requires further analysis.

## 5. Conclusions

Replicating the study in different geographic locations in the U.S. and other countries with a different health care payment system, with a focus on tightening the inclusion criteria, expanding determinants, and clarifying concepts, is warranted. Studies focusing on inclusion criteria should specify the caregiving length of time and should recruit caregivers during hospitalization, or when the caregiver provides care related to a new ADL/IADL need, in order for the baseline to be consistent. Expanding determinants should broaden the tasks and needs determinants to include medical and nursing tasks that the community dwelling elder requires to remain in the home. A phenomenological study clarifying religion and spirituality would assist in clarifying how caregivers view these two distinct but related concepts.

## Figures and Tables

**Table 1:** Instrument information.

Instrument	Subscale & Number of Questions	Measurement	Time 1 Cronbach’s Alpha	Reference Cronbach’s Alpha
NIH Promis Global Health [27]	Physical Health (PH) N = 4	1 = best 5 = worst	0.81	0.81
	Mental Health (MH) N = 4		0.85	0.86
Kosloski’s Measure of Caregivers Beliefs and Attitudes [29]	Family Relationships: Affection for Elder N = 6	1 = not true 5 = completely true	0.96	0.90
	Family Relationships: Obligation to Care N = 6		0.94	0.78
	Family Relationships: Family Values N = 8		0.80	0.74
	Attitudes Regarding Help: Spirituality N = 3	1 = strongly disagree 4 = strongly agree	0.89	0.84
Walker’s Health Promoting Lifestyle Profile II [28]	Health-Promoting Lifestyle (Health Promotion) N = 52	1 = never 4 = routinely	0.95	0.92
	Health Responsibility N = 9		0.79	0.81
	Physical Activity N = 8		0.89	0.81
	Nutrition N = 9		0.75	0.76
	Spiritual Growth N = 9		0.89	0.90
	Interpersonal Relations N = 9		0.84	0.80
	Stress Management N = 8		0.85	0.70
Hileman’s Home Caregiving Need Survey [31]	Needs Involving Information N = 14	Each question answered with 1–3 and 1–7 (1) Not applicable (2) Needs involving information (3) How satisfied is this need for you 1 = not very important 7 = very important	0.91	0.96
	Needs Involving Your Household N = 14		0.92	0.95
	Patient Care Needs (energy) N = 16		0.91	0.93
	Personal Needs (rest) N = 11		0.88	0.88
	Spiritual Needs (hope) N = 6		0.84	0.87
	Psychological Needs (stress) N = 30		0.96	0.93
Oberst’s Caregiving Burden Scale [30]	Demand N = 15	1 = great deal 5 = none	0.92	0.83
	Difficulty N = 15		0.94	0.89

Items in parentheses entered into regression analysis.

**Table 2:** Participant characteristics (Time 1).

		**N**	**Mean**	**Standard Deviation**	**Range**
Age	Male	11	68	15.94	24–86
	Female	60	59	8.90	38–77
Number of hours of care provided per week	Male	10	130	65.00	0–168
	Female	58	95	69.18	0–168
Number of Activities of Daily Living (ADL) provided	Male	11	2.55	1.86	0–5
	Female	63	2.71	1.78	0–5
Number of Independent Activities of Daily Living (IADL) provided	Male	11	6.55	1.57	4–8
	Female	63	9.76	1.20	4–8
Total number of ADL/IADL provided	Male	11	9.09	2.84	5–13
	Female	63	9.48	2.58	4–13
Length of time caregiving	Male	10	5 years	2.86	3 months–20 years
	Female	60	5 years	0.48	2.5 months–30 years
			**N (Percent)**		
Number of Care recipients with medical diagnosis Alzheimer/dementia	Male		6 (16.7%)		
	Female		30 (83.3%)		
Number of caregivers with chronic health condition (DM, CVD, Respiratory or musculoskeletal problems)	Male		7 (16.3%)		
	Female		36 (83.7%)		

**Table 3:** Predictors associated with PH and MH by time; stepwise backwards linear regression final model for each time point.

Variable	Coefficient					
	Baseline		8 Weeks		16 Weeks	
	PH	MH	PH	MH	PH	MH
Health Promotion Health Responsibility					−0.90 **	
Health Promotion and Physical Activity	0.32 **				0.32 **	
Health Promotion Nutrition					0.32 *	
Health Promotion and Spiritual Growth	0.48 **	0.38 **	0.37 **	0.44 **	0.47 **	0.40 **
Health Promotion Interpersonal relations		0.45 *				
Demand						
Difficulty						
Family values affection						
Family values obligation						
Family relations values						
Attitudes regarding help spirituality						
Needs		−0.03 **	−0.02 *	−0.05 **		−0.03 **

Significance: * <0.05; ** <0.01.

**Table 4:** Predictors associated with PH and MH in longitudinal analysis; generalized estimating equations (GEEs).

Variable	Physical Health		Mental Health	
	Coefficient (CI)	*p*-Value	Coefficient (CI)	*p*-Value
8 week	−0.06 (−0.20, 0.08)	0.38	−0.19 (−0.34, −0.05)	<0.01 **
16 week	−0.07 (−0.20, 0.06)	0.29	−0.28 (−0.44, −0.12)	<0.01 **
Health Responsibility	−0.18 (−0.35, −0.01)	0.04 *	0.08 (−0.15, 0.30)	0.51
Physical Activity	0.18 (0.05, 0.32)	<0.01 **	0.05 (−0.08, 0.19)	0.44
Nutrition	0.21 (0.02, 0.40)	0.03 *	−0.03 (−0.027, 0.21)	0.79
Spiritual Growth	0.27 (0.04, 0.5)	0.02 *	0.26 (0.01, 0.50)	0.04 *
Interpersonal Relations	0.08 (−0.12, 0.28)	0.43	0.13 (−0.17, 0.43)	0.39
Stress Management	−0.04 (−0.26, 0.19)	0.74	0.18 (−0.11, 0.34)	0.31
Needs	−0.01 (−0.03, 0)	0.05 *	−0.03 (−0.05, −0.02)	<0.01 **

Significance: * <0.05; ** <0.01.

**Table 5:** Change in health.

Dependent Variable	Time		Mean		Confidence Interval
Physical Health	baseline	2	0.09364	1.000	−0.1883, 0.3755
		3	0.19843	0.272	−0.0835, 0.4803
	8 weeks	1	−0.09364	1.000	−0.3755, 0.1883
		3	0.10480	1.000	−0.1860, 0.3956
	16 weeks	1	−0.19843	0.272	−0.4803, 0.0835
		2	−0.10480	1.000	−0.3956, 0.1860
Mental Health	baseline	2	0.24773	0.222	−0.0851, 0.5806
		3	0.36515 *	0.026 *	0.0323, 0.6980
	8 weeks	1	−0.24773	0.222	−0.5806, 0.0851
		3	0.11742	1.000	−0.2259, 0.4607
	16 weeks	1	−0.36515 *	0.026 *	−0.6980, −0.0323
		2	−0.11742	1.000	−0.4607, 0.2259

Significance: * <0.05.
